# Cerebral Edema Formation After Stroke: Emphasis on Blood–Brain Barrier and the Lymphatic Drainage System of the Brain

**DOI:** 10.3389/fncel.2021.716825

**Published:** 2021-08-16

**Authors:** Sichao Chen, Linqian Shao, Li Ma

**Affiliations:** Department of Neurosurgery, Sir Run Run Shaw Hospital, Zhejiang University School of Medicine, Hangzhou, China

**Keywords:** cerebral edema, blood-brain barrier, glymphatic system, meningeal lymphatic system, ischemic stroke

## Abstract

Brain edema is a severe stroke complication that is associated with prolonged hospitalization and poor outcomes. Swollen tissues in the brain compromise cerebral perfusion and may also result in transtentorial herniation. As a physical and biochemical barrier between the peripheral circulation and the central nervous system (CNS), the blood–brain barrier (BBB) plays a vital role in maintaining the stable microenvironment of the CNS. Under pathological conditions, such as ischemic stroke, the dysfunction of the BBB results in increased paracellular permeability, directly contributing to the extravasation of blood components into the brain and causing cerebral vasogenic edema. Recent studies have led to the discovery of the glymphatic system and meningeal lymphatic vessels, which provide a channel for cerebrospinal fluid (CSF) to enter the brain and drain to nearby lymph nodes and communicate with the peripheral immune system, modulating immune surveillance and brain responses. A deeper understanding of the function of the cerebral lymphatic system calls into question the known mechanisms of cerebral edema after stroke. In this review, we first discuss how BBB disruption after stroke can cause or contribute to cerebral edema from the perspective of molecular and cellular pathophysiology. Finally, we discuss how the cerebral lymphatic system participates in the formation of cerebral edema after stroke and summarize the pathophysiological process of cerebral edema formation after stroke from the two directions of the BBB and cerebral lymphatic system.

## Introduction

Worldwide, stroke is the leading cause of adult disability and the second main cause of death after coronary heart disease, affecting more than 13.7 million patients every year (GBD 2015 Mortality and Causes of Death Collaborators, 2016; Phipps and Cronin, 2020). Stroke includes ischemic stroke, subarachnoid hemorrhage (SAH), and cerebral hemorrhage, of which ischemic stroke accounts for 80% of all stroke cases (GBD 2015 Mortality and Causes of Death Collaborators, 2016). They are all accompanied by cerebral edema, and swollen tissues in a fixed volume of the skull caused by edema that exert a mechanical force on adjacent tissues and capillaries, leading to decreased blood perfusion, aggravated ischemia and edema, and tissue damage (Simard et al., 2007; Rungta et al., 2015; Leinonen et al., 2017). Malignant cerebral edema is a devastating complication of ischemic infarction, which accounts for 10% to 78% of patients with all types of ischemic stroke (Wu et al., 2018). It can result in massive cerebral swelling, subsequent raised intracranial pressure (ICP), rapid neurological deterioration, and transtentorial herniation (Huttner and Schwab, 2009). The mortality rate in patients with malignant cerebral edema is close to 80% (Huttner and Schwab, 2009). However, treatment options for cerebral edema remain limited, and available treatments are suboptimal. Therefore, understanding the underlying molecular and cellular mechanisms of edema formation is critical.

Cerebral edema occurs in three distinct phases that mature separately over time and space: the early cytotoxic edema phase, the subsequent ionic edema phase, and the most severe vasogenic edema phase (Simard et al., 2007; Stokum et al., 2016; Clément et al., 2020). Cytotoxic edema occurs within minutes after ischemic insult without BBB disruption, which is usually the consequence of ATP depletion and is characterized by the swelling of astrocytes and neuronal dendrites (Liang et al., 2007; Risher et al., 2009; Badaut et al., 2011b). Although cytotoxic edema does not generate tissue swelling, the ionic gradient between the vascular compartment and interstitial fluid (ISF) it causes provides the driving force for the subsequent ionic and vasogenic edema, which do cause swelling (Mori et al., 2002; Simard et al., 2007; Stokum et al., 2016). The term ionic edema (interstitial edema) was introduced to explain the form of cerebral edema in the early hours of ischemic stroke, with barrier breakdown not occurring until 4–6 h after the onset of ischemia (Gotoh et al., 1985; Young et al., 1987; Hatashita and Hoff, 1990; Schielke et al., 1991; Simard et al., 2007). The ionic edema is followed by BBB breakdown: vasogenic edema, which is characterized by allowing water and plasma proteins, such as albumin and IgG, to leak into the brain interstitial compartment (Stokum et al., 2016; Zhang et al., 2020). The stepwise recruitment of transcellular and paracellular pathways contributes to the breakdown of the BBB (Dreier et al., 2018). As early as 6 h after stroke, a rise in the number of caveolae and an increased transcytosis rate disturb the transcellular pathway, whereas structural abnormalities in tight junctions (TJs) activate the paracellular pathway after 2 days (Kang et al., 2013; Knowland et al., 2014).

The water source of ionic brain edema can only come from blood and CSF (Simard et al., 2007). The hypothesis that local blood perfusion acts as a water source for ionic cerebral edema has been confirmed in numerous experiments. For example, the post-ischemic degree of reperfusion is positively correlated to edema (Bell et al., 1985). Furthermore, edema fluid is first found and located mostly in peri-infarct regions that are actively perfused (Quast et al., 1993; Simard et al., 2006). Recent studies describe that a brain-wide paravascular pathway provides a conduit for CSF influx prompted us to ponder whether CSF serves as the immediate source of ions and water for edema (Iliff et al., 2012). The hypothesis that CSF influx can drive ionic brain edema formation is supported by some indirect evidence (Thrane et al., 2014). For example, the increased paravascular space following pericyte constriction and microvascular collapse can reduce resistance to CSF influx (Hall et al., 2014). Furthermore, the impairment of glymphatic pathway function after injury or infarction is likely to trigger a reduced clearance of interstitial solutes and exacerbate edema (Ren et al., 2013; Iliff et al., 2014). This circumstantial evidence is not convincing. However, Humberto Mestre and his colleagues directly described that the influx of CSF into the brain tissue drives acute tissue swelling within minutes of ischemic stroke (Mestre et al., 2020). The discovery of the classical lymphatic drainage system in the dura mater of the brain, which can absorb CSF from the adjacent subarachnoid space and provides the pathway for the entrance and exit of immune cells from the CNS, calls for a reassessment of cerebral edema formation and sheds new light on the etiology of the neuroinflammatory mechanisms of BBB damage in ischemic stroke (Aspelund et al., 2015; Louveau et al., 2015).

Several fundamental pathophysiologic processes contribute to cerebral edema development after stroke, including the disruption of TJs, the loss of homeostatic ionic gradients, inflammatory responses, and the activated glymphatic system. After the disruption of TJs, inflammatory responses and the activation of ion channels can be considered to promote the occurrence of cerebral edema by exerting an influence on the permeability of BBB. We summarize these aspects into only one part: the increase of BBB permeability to promote cerebral edema. However, the influence of the CNS lymphatic system on the occurrence and progression of cerebral edema induced by stroke has not been reviewed. Therefore, this article summarizes the various pathophysiologic processes that affect the permeability of the BBB to promote the occurrence of cerebral edema and focuses on the effect of the CNS lymphatic system on the development of cerebral edema after stroke.

## Increased BBB Permeability Contributes to Edema

BBB dysfunction that occurs during cerebral ischemia enables considerable vascular fluid to pass through microvascular endothelium into the brain interstitial compartment and eventually leads to vasogenic edema formation (Sandoval and Witt, 2008; Prakash and Carmichael, 2015; Stokum et al., 2016). Any disorder of the factors that maintain the functional integrity of the BBB will lead to an increase in the permeability of the BBB. Here, we discuss and summarize the mechanisms that can increase the permeability of the BBB. In theory, these mechanisms will eventually lead to the aggravation of cerebral edema after stroke.

### Anatomical Considerations of BBB

**FIGURE 1:**
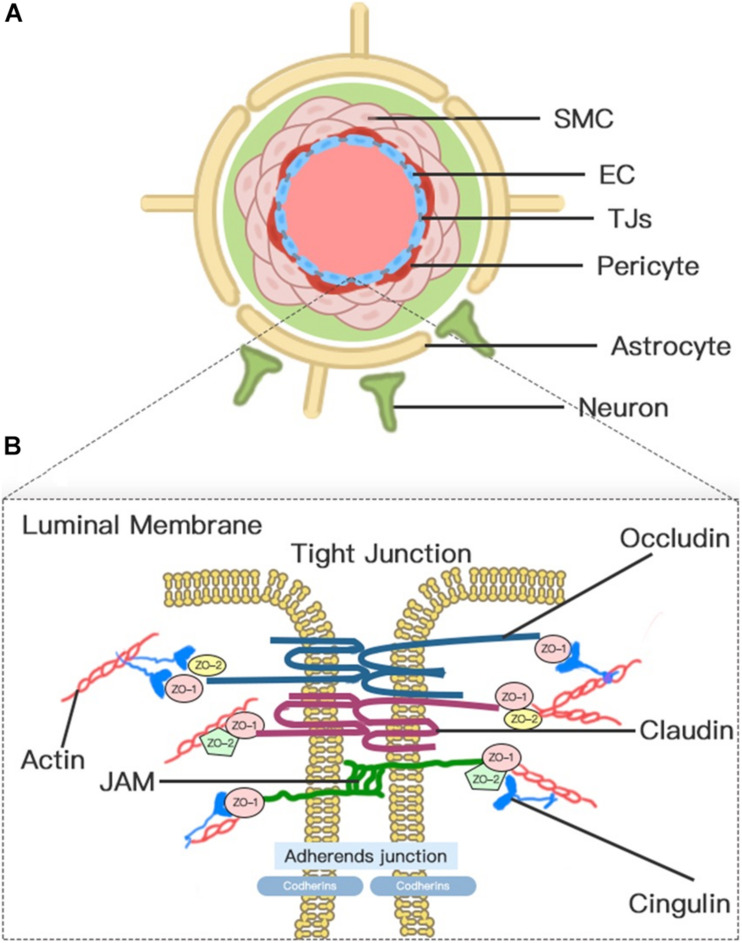
Neurovascular unit (NVU) and blood–brain barrier (BBB). **(A)** Transverse-section representation of the NVU. The concept of the NVU highlights the importance of the intimate interactions between components of the BBB and cells in brain parenchyma, including pericytes, astrocytes, microglia, and neurons. The BBB is centrally positioned within the NVU, which is formed by a monolayer of endothelial cells sealed by tight junctions. **(B)** A schematic blow-up of the tight junctions (TJs) and adherens junctions (AJs) at the BBB as defined in the text.

The concept of BBB was first proposed in the early 20th century (Zlokovic, 2008). Today, the concept of the BBB as an impermeable barrier has evolved into a dynamic and metabolic interface that maintains the fragile homeostasis of the brain through regulating the trafficking of fluid and solutes bi-directionally and metabolizing potentially neurotoxic compounds (Jiang et al., 2018). Although the BBB is formed primarily by the brain microvascular endothelium, the complete function of the BBB requires the harmonious functional interplay of multiple cells (Figure 1), including astrocytes, pericytes, microglia, neurons, vascular smooth muscle cells (SMCs), and extracellular matrix (ECM) components. Therefore, to emphasize further the cellular interplay in maintaining the function of the BBB, we introduce the concept of the neurovascular unit (NVU).

Blood–brain barrier endothelial cells (ECs), which act as the first line of defense in the innermost layer of the BBB, are differentiated from common ECs by a lack of fenestrations, minimal pinocytotic activity, and the presence of extensive TJs and numerous mitochondria (Abdullahi et al., 2018). Notably, the brain endothelium plasmalemma is divided into luminal and abluminal membrane faces by extensive inter-endothelial TJs. The different expression of transport proteins and metabolic enzymes between luminal and abluminal membrane faces leads to the polarization of ECs and finally restricts the flux of blood-to-brain substances across the microvascular endothelium (Sanchez-Covarrubias et al., 2014). The constitutive and *de novo* expression of ion transporters serve as drivers of ionic edema after stroke (Stokum et al., 2016). Several factors that are produced by ECs, such as platelet-activating factor, superoxide radicals, endothelins, and eicosanoids, impair perfusion, increase BBB permeability, and induce cell damage when overexpressed (Spatz, 2010).

Pericyte is a mesenchymal cell type located in the endothelial basement membrane of the capillaries and microvessels (Spatz, 2010). Astrocytes, whose endfeet are almost surrounding the abluminal ECs surface, act as intermediaries in the NVU responding to neuronal synaptic activity (Koehler et al., 2006). Pericytes and astrocytes all play important roles in the formation, maturation, and maintenance of the BBB and the regulation of capillary blood flow (Alvarez et al., 2013; Stokum et al., 2016; Jiang et al., 2018). Pericyte-deficient mice are identified with endothelial hyperplasia, increased capillary diameter, an abnormal cellular distribution of junctional proteins, and increased transendothelial permeability (Hellstrom et al., 2001). Chemical factors produced by astrocytes, such as Sonic Hh, vascular endothelial growth factor (VEGF), angiopoietins-1, Src suppressed C kinase substrate, and TGF-β, can promote vascular growth and BBB differentiation and maturation and support BBB integrity (Alvarez et al., 2011, 2013). In addition, a high density of aquaporin-4 (AQP4) water channels highly polarized to perivascular astrocytic endfeet act as an indispensable component of the glymphatic system, which facilitates the circulation of CSF through the brain interstitial space (Iliff et al., 2012).

Endothelial cell junctions include TJs and adherens junctions (AJs) (Figure 1). TJs between adjacent ECs are responsible for the formation of a continuous and impermeable barrier. AJs are likely to play an auxiliary role to help the localization and stabilization of TJs that are formed by cadherins and associated proteins that are directly linked to actin filaments (Dejana et al., 2008; Redzic, 2011). Three integral transmembrane proteins, namely, claudins, occludin, and junctional adhesion molecules (JAMs), are involved in the assembly of TJs (Figure 1; Stamatovic et al., 2016). The stability of TJs can be maintained by cytoplasmic accessory molecules comprising zonula occludens (ZO)-1, ZO-2, and ZO-3, and cingulin, which fasten these transmembrane proteins to the actin cytoskeleton (Ballabh et al., 2004). In addition to these cellular components, the luminal membrane and basement membrane also participate in maintaining vascular permeability (Spatz, 2010). The glycocalyx coating the luminal EC membrane is composed of proteoglycans, glycosaminoglycan, and absorbed plasma proteins, and glycocalyx damaged by ischemia or injury permits the attachment of leukocytes (Spatz, 2010). The basement membrane separates the endothelium from the astrocyte and prevents the vascular leakage of plasma protein through the cooperation of multiple ECM proteins, including collagens, laminins, heparin sulfate proteoglycans, fibronectin, vitronectin, nidogens, perlecan, and agrin (Spatz, 2010; Yao and Tsirka, 2014).

### Pathways Involved in BBB Permeability and Edema

#### TJ Disruption

**FIGURE 2:**
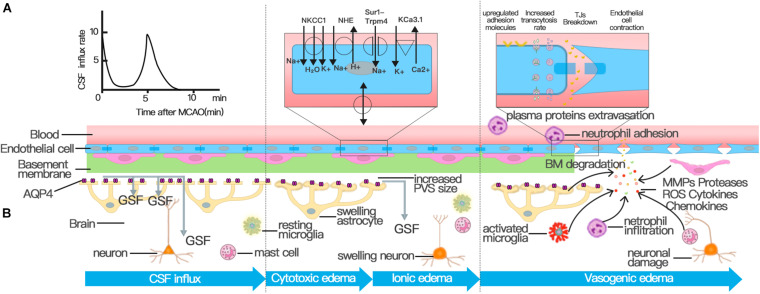
Three distinct phases of cerebral edema. **(A)** CSF influx occurred and peaked at 11.4 ± 1.8 s and 5.24 ± 0.48 min after MCAO. The first peak of CSF influx is the hydrostatic pressure gradient due to vascular obstruction. The increased PVS size triggered by spreading depolarizations (SDs) drives the second wave of perivascular CSF influx, which facilitates the swelling of astrocytic endfeet. **(B)** Cerebral edema can be classified into three phases: cytotoxic, ionic, and vasogenic edema phases. The cytotoxic edema phase is characterized by swelling of astrocytes and happens simultaneously with the second peak of CSF entry. The second stage of cerebral edema formation, the ionic edema stage, is mainly driven by endothelial ion channels and transporters in the context of an intact BBB, such as NKCC, NHE, KCa3.1, and Sur1-Trpm4 channel. The breakdown of BBB causes vasogenic edema. Successive alterations to the transcellular and paracellular pathway of the BBB contribute to the breakdown of BBB following stroke. First, the increase in the number of endothelial caveolae and the rate of transcytosis impairs BBB function by disturbing the transcellular pathway. Then the destruction of TJs activates the paracellular pathway. In addition to NVU cells involved in the regulation of BBB permeability, various immune cells and inflammatory factors play an important role in the destruction of TJs.

Vasogenic edema is characterized by increased paracellular permeability of the BBB, which is mainly caused by TJ disruption (Figure 2; Wolburg and Lippoldt, 2002; Stokum et al., 2016). Therefore, the pathological mechanism of TJ destruction is particularly important. Progressive TJ dysfunction can be organized into three phases: protein modification, protein translocation, and protein degradation; each of the phases can increase BBB permeability and promote the formation of edema (Jiang et al., 2018). In the first phase, inflammatory factors and cytokines released during ischemic brain injury, such as VEGF, chemokine monocyte chemoattractant protein-1 (CCL2), tumor necrosis factor (TNF)-α, and IL-6, can induce the phosphorylation of TJs, leading to BBB hyperpermeability (Stamatovic et al., 2006; Murakami et al., 2009; Murakami et al., 2012; Rochfort and Cummins, 2015). Attenuating TJ phosphorylation can also inhibit the leakage of BBB after transient focal cerebral ischemia (Kago et al., 2006; Takenaga et al., 2009). In the second phase, TJ translocation, which is largely mediated by endocytosis and actin polymerization, also compromises BBB integrity (Jiang et al., 2018). For example, occludin, claudin-5, and JAM-A redistribute from the cytoskeleton or interendothelial cell cleft after ECs are treated with CCL2 or cultured in an environment with oxygen glucose deprivation (OGD) (Stamatovic et al., 2009, 2012; Liu et al., 2012). Experiments have also identified the redistribution of occludin and cadherin from the membrane fraction to the actin cytoskeleton fraction due to robust actin polymerization and stress fiber formation (Shi et al., 2016). The degradation of TJ protein is the last step of TJ disruption and the most critical step to destroy the integrity of BBB, which causes increased paracellular leakage and the infiltration of peripheral immune cells at the BBB. The activation of matrix metalloproteinases (MMPs) is one of the most significant contributors to TJ degradation in stroke (Abdullahi et al., 2018). MMP-2 and MMP-9 are the most studied and main MMPs that are increased following stroke (Turner and Sharp, 2016). Although ECs, microglia, and astrocytes overexpress MMPs, infiltrating neutrophils have proved to be a major source of MMP-9 (Romanic et al., 1998; Tang et al., 2006; Turner and Sharp, 2016). Upregulated MMP-9 and MMP-2 after stroke degrade TJs, such as occludin and Claudin-5, and microvascular basal lamina (Asahi et al., 2001; Liu et al., 2012; Qi et al., 2016). By selectively inhibiting MMP-2/9, the impairment of BBB integrity and the volume of edema in cerebral ischemic mice can be significantly reduced (Cui et al., 2012; Liu et al., 2012; Qi et al., 2016). Therefore, the destruction of TJs, which leads to the decrease of BBB integrity, is one of the key factors leading to the formation of cerebral edema after stroke.

#### Actived NVU Cells

The NVU, which emphasizes cell–cell and cell–matrix interactions in the brain, provides a more integrative answer to BBB disruption after stroke (Lo et al., 2003). The permeability of the BBB is constantly regulated by different cell types in the NVU. Therefore, the occurrence of cerebral edema after stroke is also closely related to various cellular components in the NVU.

As a first-line defense located between the blood plasma and interstitium, the continuous endothelium is essential for physiologic homeostasis and directly reacts to harmful substances from the periphery. Under the pressure of ischemia and hypoxia after stroke, actin polymerization elicits stress fibers and concomitant endothelial cell contraction mediated by zipper-interacting protein kinase (ZIPK) through the phosphorylation of the myosin light chain (Vandenbroucke et al., 2008; Komarova and Malik, 2010; Shi et al., 2016; Zhang et al., 2019). Following endothelial cell contraction, the formed paracellular gap improves paracellular permeability and allows macromolecules and inflammatory cells to enter brain parenchyma (Komarova and Malik, 2010; Zhang et al., 2019). The global deletion of ZIPK in an animal model of middle cerebral artery occlusion (MCAO) significantly attenuates BBB dysfunction by inhibiting EC contraction, as proven by reduced infarct and edema volume (Zhang et al., 2019). Oxidative stress after stroke can also induce endothelial cell apoptosis, which is suggested to have detrimental consequences on BBB integrity, subsequently leading to brain edema (Rizzo and Leaver, 2010; Al Ahmad et al., 2012). Protecting ECs from apoptosis after stroke is beneficial to the integrity of BBB and the reduction of brain edema (Park et al., 2004; Zhang et al., 2016; Yang et al., 2017). The increased number of endothelial caveolae and transcytosis rate account for the BBB disruption that occurs in the early phase of stroke (Knowland et al., 2014; Nahirney et al., 2016; Haley and Lawrence, 2017).

Activated pericytes and astrocytes also contribute to the breakdown of BBB and promote cerebral edema formation after stroke. Pericytes migrate from the brain microvascular wall in a rat MCAO model, and the detachment increases the permeability of the BBB to water and tracers (Gonul et al., 2002; Duz et al., 2007; Armulik et al., 2010). Stimulation by some factors, such as TNF-α and thrombin, makes pericytes and astrocytes important sources of MMP-9, which can cause BBB dysfunction through the degradation of TJs and the basal lamina and the enhancement of pericyte migration (Takata et al., 2012; Machida et al., 2015; Turner and Sharp, 2016). Activated astrocytes in stroke can also facilitate the destruction of BBB by increasing VEGF (Li et al., 2014). In EC–astrocyte co-cultures, microvesicles released from ECs cultured in OGD conditions promote the apoptosis of astrocytes, increase the permeability of BBB, and downregulate TJ proteins (Pan et al., 2016).

#### Inflammation Responses

After stroke onset, circulating leukocytes adhere, migrate, and eventually accumulate in the lesion site and then release inflammatory factors to cause secondary BBB disruption. Neutrophils are the earliest leukocyte subtype that infiltrates an ischemic brain and contribute to the breakdown of BBB by secreting MMP-9, neutrophil elastase, and reactive oxygen species (ROS) (Figure 2; Jickling et al., 2015). In a study of a mouse model of ischemic stroke, we proved that MMP9, which is derived mainly from neutrophils rather than brain parenchymal cells, causes BBB disruption (Gidday et al., 2005; Tang et al., 2006; Wang et al., 2009). In an experimental intracerebral hemorrhage, neutrophil depletion by anti-polymorphonuclear leukocyte antibodies reduces the production of MMP-9, infiltration of activated microglia/macrophages, and leakage of the BBB (Moxon-Emre and Schlichter, 2011). Neutrophil elastase released from neutrophils is another harmful inflammatory reaction that contributes to BBB disruption and vasogenic edema (Stowe et al., 2009; Ikegame et al., 2010). Neutrophils exacerbate BBB breakdown by producing neutrophil extracellular traps (Kang et al., 2020), which damage ECs by releasing many cytotoxic proteases, such as histone, elastase, and myeloperoxidase (Villanueva et al., 2011). In addition to that of neutrophils, the recruitment of monocytes and lymphocytes is also involved in the regulation of BBB function. T cells and B cells have both protective and damaging roles in cerebral ischemia; however, the role of each type of lymphocyte in stroke and the effect on BBB permeability after ischemic stroke should be further clarified (Jian et al., 2019; Yang et al., 2019).

Mast cells, as resident cells in the brain and meninges, also promote BBB damage and edema formation by releasing their granule contents, such as histamine, TNF-α, proteases, heparin, and various chemoattractants (Figure 2; Lindsberg et al., 2010; Arac et al., 2014; Dong et al., 2014). Rats treated with a mast cell stabilizer (cromoglycate) after MCAO show significantly reduced ischemic brain swelling by 40%, BBB leakage by 50%, and less postischemic neutrophil infiltration by 37% (Strbian et al., 2006).

Macrophages, which can be transformed from brain-inhabited microglia or differentiated from peripheral monocyte, also promote neuroinflammation and blood vessel disintegration after ischemic stroke (da Fonseca et al., 2014; Benakis et al., 2015; Fumagalli et al., 2015; Jian et al., 2019). Both microglia- and monocyte-derived macrophages have a phagocytic function, express the same phenotypic markers, and can transform to pro-inflammatory/anti-inflammatory (M1/M2) phenotype. The number of monocytes infiltrating the ischemic brain is lower than that of activated microglia (Kokovay et al., 2006; Denes et al., 2007). Therefore, we mainly discuss the damaging effect of microglia on BBB. Recently, the presence of CD31-positive particles (blood vessels) in the intracellular vesicles of perivascular microglia indicates the phagocytosis of blood vessels by perivascular microglia and finally contributes to the breakdown of the BBB (Jolivel et al., 2015). Ischemia can also induce the generation of NOX-dependent ROS in brain microglia and inflict damage on BBB by activating transcription factors or ion channels, such as JNK, p38 MAPK, JAK-STAT, NF-kB, and Hv1 (Kacimi et al., 2011; Wu et al., 2012). The expression of a large array of inflammatory mediators, such as IL-1, IL-6, matrix metalloproteases, MMPs, and TNF-α, by microglia after ischemic stroke also enhances vascular permeability in the brain (Zhou et al., 2013; Lee et al., 2014; Thurgur and Pinteaux, 2019). The inhibition of microglial activation by pretreatment with minocycline can suppress vasogenic edema and infarct formation in ischemic stroke (Tanaka et al., 2018).

Various chemical factors, such as cytokines, chemokines, ROS, MMPs, and VEGF, secreted from the periphery and resident immune cells and glial cells play a key role in the regulation of BBB disruption in ischemic stroke (Yang et al., 2019). Among the most extensively studied cytokines in the context of stroke, TNF-α, IL-1, and IL-6 have been shown to disrupt the BBB (Blamire et al., 2000; Pradillo et al., 2012; Cohen et al., 2013; Rochfort et al., 2014; Kangwantas et al., 2016; Pradillo et al., 2017). The most notorious upregulated chemokines in response to hypoxia/ischemia are CCL2, macrophage inflammatory protein-1α, and stromal-derived factor-1, which play an important role in leukocyte recruitment and promote BBB destruction (Eugenin and Berman, 2003; Stamatovic et al., 2005; Dimitrijevic et al., 2006; Chui and Dorovini-Zis, 2010; Takata et al., 2012). Oxidative stress caused by ROS and nitric oxide (NO) plays a critical role in MMP activation and BBB breakdown after stroke (Yang et al., 2019). Leukocytes, glial cells, and vascular ECs are important sources of ROS and NO (Iadecola et al., 1995; Lassegue and Clempus, 2003). Furthermore, VEGF secreted by neurons, astrocytes, and macrophages is also involved in the activation of MMPs and BBB disruption following ischemia (Kovacs et al., 1996; Valable et al., 2005; Lee et al., 2007). The upregulation of cell adhesion molecules, such as selectins, immunoglobulin superfamily, and integrins, promotes the infiltration of leukocytes, especially neutrophils, to the CNS and leads to BBB damage (Yang et al., 2019). In general, the neuroinflammatory mechanism of BBB damage in ischemic stroke is very complex but is a promising target to reduce BBB damage, edema, and brain injury after stroke.

#### Ion Transporter Dysfunction

The depletion of intracellular ATP leads to the cytotoxic edema of all CNS cell types after ischemic stroke, of which astrocytes are particularly prominent; cytotoxic edema ultimately provides the driving force for ionic edema, vasogenic edema, and complete hemorrhagic conversion (Liang et al., 2007; Stokum et al., 2016). The mechanism behind cytotoxic edema and ionic edema is ion transporter dysfunction at the NVU rather than the breakdown of the BBB (Figure 2; Brillault et al., 2008; Stokum et al., 2016; Sifat et al., 2019).

Na + –K + –2Cl -cotransporter (NKCC) is expressed the astrocytes, neurons, and ECs of the brain (Kahle et al., 2008; Jayakumar and Norenberg, 2010), and reside predominantly in the luminal membrane of BBB ECs (O’Donnell et al., 2004). NKCC is activated via phosphorylation in response to hypoxia, aglycemia, and arginine vasopressin and contributes to edema formation during cerebral ischemia (Yan et al., 2001; Foroutan et al., 2005; Brillault et al., 2008; Yuen et al., 2014). The inhibitory effect of bumetanide on NKCC activities can reduce brain Na absorption and edema formation in rat MCAO stroke models (Yuen et al., 2014, 2019). The sodium–hydrogen antiporter (NHE) family member, NHE1, is ubiquitously expressed in all cell types in the brain; is stimulated by hypoxia, aglycemia, and arginine vasopressin as with NKCC; and contributes to astrocyte swelling, ionic edema formation, microglial activation, and BBB breakdown (Lam et al., 2009; Stokum et al., 2016; Begum et al., 2018; Song et al., 2018). The inhibition of NHE activities by the intravenous delivery of Na/H exchange inhibitor HOE642 decreases brain edema in an ischemic stroke model (Lam et al., 2009; Yuen et al., 2014, 2019). Mice with the selective ablation of the NHE1 gene in astrocytes exhibit less edema, reduced BBB breakdown, and alleviated disruption of TJ protein after transient MCAO (tMCAO) (Begum et al., 2018). Furthermore, ischemia also induces the *de novo* expression of the sulfonylurea receptor 1–transient receptor potential 4 channel (SUR1-TRPM4) in all cells of the NVU and contributes to the formation of ionic edema (Simard et al., 2014; Stokum et al., 2016). The blockage of the SUR1-TRPM4 channel results in a significant reduction in infarct volume, cerebral edema, and hemispheric swelling in rodent models of ischemic stroke (Simard et al., 2006, 2009; Wali et al., 2012). In the mouse edema model, the up-regulated SUR1-TRPM4 in astrocytes can synergize with APQ4 to promote the influx of water and the swelling of astrocytes (Stokum et al., 2018). The recently discovered KCa3.1, a calcium-activated potassium channel expressed by ECs, is also involved in the formation of cytotoxic edema after ischemic stroke (Chen et al., 2015).

Ion channels can not only cause edema by transporting ions but also participate in the activation of resident cells in the brain, such as astrocytes and microglia, and lead to the destruction of the BBB. The stimulation of NHE1 in astrocytes causes a robust release of glutamate and the pro-inflammatory cytokines interleukin (IL)-1β, IL-6, TNF-α, and MMP-9 (Cengiz et al., 2014; Begum et al., 2018). The pharmacological inhibition or genetic knockout of astrocytic NHE1 protein significantly reduces cerebral microvessel damage, BBB breakdown, and loss of the TJ protein occludin in ischemic brain (Cengiz et al., 2014; Begum et al., 2018). The Na^+^–Ca^2+^ exchanger (NCX) in astrocytes is also involved in Ca2 + induced ROS production, DNA ladder formation, and nuclear condensation (Matsuda et al., 2001). Microglial NCX, Kv1.3, and NHE1 channels all contribute to proinflammatory microglial activation (Rangaraju et al., 2017; Song S. et al., 2020). By contributing to excessive hydrogen ion extrusion and sustained NOX activation, activated NHE1 causes the production of ROS and the expression of cytokines in microglia after lipopolysaccharide or hypoxia stimulation (Liu et al., 2010; Wu et al., 2012; Lam et al., 2013). The pharmacological inhibition or genetic knockout of microglial NHE1 and Kv1.3 reduces the secretion of pro-inflammatory cytokines, such as IL-1β, IL-6, TNF-α, and iNOS (Liu et al., 2010; Shi et al., 2011; Nguyen et al., 2017; Rangaraju et al., 2017; Di Lucente et al., 2018; Song et al., 2018). NCX1-mediated Ca2 + influx plays a critical role in microglial phagocytic activity through Ca2 + -mediated purinergic receptors (Sunkaria et al., 2016). Calcium overload increases brain ROS levels in type 5 NOX-dependent manner, which contributes to BBB breakdown (Casas et al., 2019).

## Pathways Involved in the Glymphatic System and Edema

At the periphery, impaired lymphatic system function is one of the common causes of edema and leads to the pathologic accumulation of protein-rich lymphatic fluid in the intercellular interstitium (Cho and Atwood, 2002). Acquired lymphedema caused by axillary lymph node dissection and filariasis is the most common cause of clinical lymphedema (Cho and Atwood, 2002). For a long time, due to the delayed discovery of the lymphatic drainage system of the brain, research on the mechanism of cerebral edema has mainly focused on BBB. However, discoveries of recent studies, including the brain pseudolymphatic system—glymphatic system and meningeal lymphatic system—have brought light to us to clarify the mechanism of cerebral edema.

### Anatomical Considerations of the Glymphatic System

**FIGURE 3:**
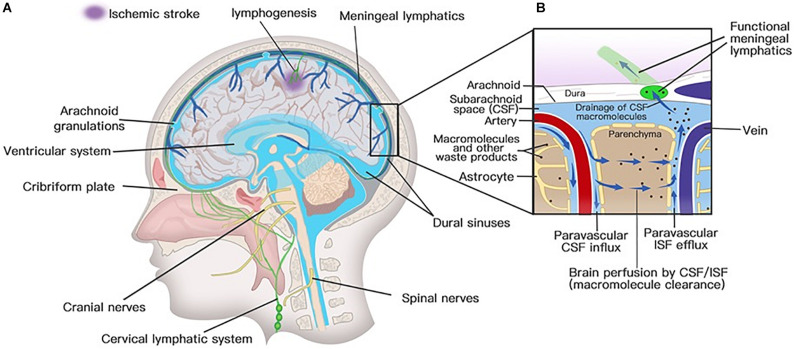
Lymphatic drainage system in the brain. **(A)** Meningeal lymphatic vessels run down toward the base of the skull along the sinus, the vein, and the meningeal arteries and drain out of the skull via the foramina of the base of the skull alongside arteries, veins, and cranial nerves. Meningeal lymphatic cells grow into the injured brain parenchyma induced by photochemical thrombosis. **(B)** Cerebrospinal fluid (CSF) enters the parenchyma by bulk flow along paravascular spaces, and ISF is cleared along paravenous drainage pathways. Meningeal lymphatic vessels absorb CSF from the adjacent subarachnoid space and ISF from the glymphatic system and transport fluid into deep cervical LNs (dcLNs) via foramina at the base of the skull.

Solutes in CSF have been thought to recycle from the subarachnoid space into brain parenchyma by the convective bulk flow rather than via an anatomically discrete structure (Abbott, 2004; Sykova and Nicholson, 2008). The precise anatomical or functional structures for the clearance of metabolic waste products from the ISF to the CSF were first described by Iliff and his colleagues (Iliff et al., 2012). Via *in vivo* two-photon imaging and other techniques, the movement of a fluorescent tracer injected in the subarachnoid compartment flowing into and through the brain interstitium was depicted to represent the exchange of CSF and ISF. In the initial segments of the pathway, flux fluid and macromolecules from the subarachnoid space rapidly enter the brain by bulk flow through paravascular spaces called Virchow Robinson spaces, which exist around vascular SMCs and perivascular astrocytic endfeet (Figure 3; Iliff et al., 2012). Then, fluids and macromolecules accumulate around along capillaries and parenchymal venules and are eventually cleared along paravenous drainage pathways (Figure 3; Iliff et al., 2012). The phenomenon of larger tracer from the subarachnoid space being confined in paravascular spaces of the brain is also consistent with a recent study demonstrating that narrow clefts between overlapping endfeet may serve a sieving function to control the exchange of water and solutes between blood and brain (Mathiisen et al., 2010). In addition, paravascular AQP4 channels, which are highly polarized to paravascular astrocytic endfeet (Mathiisen et al., 2010), facilitate bulk ISF solute clearance from the parenchyma (Iliff et al., 2012). The putative glymphatic transports have also been successfully demonstrated in humans with BBB disruption using non-invasive, high-resolution 3D isotropic contrast-enhanced T2 fluid-attenuated inversion recovery imaging (Wu et al., 2020).

Perivascular drainage pathways refer to a route responsible for the solutes diffuse across brain ECs through basement membranes between the SMCs in the tunica media of capillaries and arteries (Carare et al., 2008; Morris et al., 2016). The perivascular drainage of ISF and solutes is in the reverse direction of blood flow, which only occurs after some form of attachment of solutes or valve-like mechanism to prevent backflow during the pulse wave (Schley et al., 2006; Carare et al., 2008; Morris et al., 2016). However, the latest research has constructed a novel multiscale model of arteries to prove that the arterial pulsations from the heart are not strong enough to be the motive force for perivascular drainage, whereas the vasomotion of cerebrovascular SMCs acts as the drivers of perivascular drainage (Aldea et al., 2019). Perivascular drainage pathways should be further elaborated because the relatively dense pericapillary basement membrane is usually considered a physical obstacle to solute movements, and the non-specific binding of the fluorescent dextran tracer to the capillary basement membrane may cause the illusion of the abovementioned results (Barber and Lieth, 1997; Brinker et al., 2014). As the study on the effect of post-stroke perivascular drainage of edema formation is very limited, this review focuses on the effects of paravascular drainage on edema formation.

### Paravascular Pathway Is Involved in Edema Formation

To test whether CSF is the source of edema fluid, Humberto Mestre and his colleagues deduced a subtle way to study changes in glymphatic function after MCAO; the approach involved the use of *in vivo* magnetic resonance and multimodal optical imaging to map the influx of CSF tagged with a fluorescent tracer (Mestre et al., 2020). They found that the influx of CSF along paravascular spaces occurred and peaked at 11.4 ± 1.8 s and 5.24 ± 0.48 min after MCAO and built a new notion that CSF is the primary source of early edema fluid after ischemic stroke (Figure 2; Mestre et al., 2020), which broke the traditional concept that CSF is not a source of edema fluid (Stokum et al., 2016). The hydrostatic pressure gradient caused by a loss in blood flow after the MCAO was used to explain the first peak in the CSF influx. Experiments also proved that the pressure gradient in distal paravascular spaces caused by spreading depolarization drove CSF influx and caused the spreading edema, which also depends on AQP4 expression (Mestre et al., 2020). Therefore, the influx of CSF in the glymphatic system is increased after ischemic stroke (Mestre et al., 2020). However, before this article was published, most articles demonstrated a decreased paravascular CSF influx after ischemic stroke (Iliff et al., 2013; Gaberel et al., 2014; Ji et al., 2021). The possible reasons for these conflicting conclusions are the different time windows of observation in each experiment and the different models constructed. For example, this study observed the change of glymphatic perfusion at 3 and 24 h after MCAO but did not study the glymphatic influx rate within 20 min (Gaberel et al., 2014). The establishment of a model of internal carotid artery ligation may also lead to different conclusions from the model of MCAO (Iliff et al., 2013). The mechanism of cerebral edema should be explored by conducting more studies to observe the change of CSF influx in the glymphatic system in each time window after stroke.

Solute clearance along paravascular spaces is also markedly impaired after ischemic stroke (Arbel-Ornath et al., 2013; Pu et al., 2019). The glymphatic system may also play a positive effect in clearing edema fluid in days and weeks after stroke (Lempriere, 2020). Therefore, future works should investigate how the function of the lymphatic system can be adjusted to optimize edema recovery.

A recent article points out that the pressure gradient caused by vasoconstriction draws the influx of CSF into the brain parenchyma, driving acute ischemic tissue swelling (Mestre et al., 2020). As such, cerebral vasospasm, which is common in SAH, may also promote early edema formation after SAH in a manner similar to ischemic stroke (Rowland et al., 2012). However, this hypothesis needs more experimental proof.

Studies have also shown severely impaired glymphatic system perfusion and a reduced glymphatic system waste clearance function from the brain parenchyma after SAH (Gaberel et al., 2014; Goulay et al., 2017; Golanov et al., 2018). The main reason for glymphatic inhibition is the occlusion of perivascular spaces by fibrin/fibrinogen clots, which can be removed through the intraventricular injection of fibrinolytic tissue-type plasminogen activator (Gaberel et al., 2014; Goulay et al., 2017). Although these trials did not provide evidence that intravascular thrombus aggravates edema after SAH by compressing the paravascular space, a recent study showed that brain edema can be alleviated by preserving the function of the glymphatic system after SAH (Fang et al., 2020). Therefore, the impaired function of glymphatic system waste clearance may be a factor leading to the formation of cerebral edema after SAH. In general, the function of the glymphatic system may play an important role in the cerebral edema of SAH. However, the specific mechanism still requires confirmation by more experimental studies.

### AQP4 Is Involved in Edema Formation

The AQP family contains 13 different members, of which the expression levels of AQP1, AQP4, and AQP9 in rodent models and humans are upregulated after stroke (Gonen and Walz, 2006; Ribeiro Mde et al., 2006; Yatsushige et al., 2007; Vella et al., 2015; Stokum et al., 2018). Since studies have determined the involvement of AQP4 in the process of cerebral edema in early 2000, AQP4 has become a hot research (Manley et al., 2000; Vella et al., 2015). However, the exact mechanism of AQP4 regulating edema is still controversial and unclear. Now, the discovery of the glymphatic system and the special status of AQP4 in the glymphatic system have brought a new understanding of these controversies (Rasmussen et al., 2018). Therefore, the role of AQP4 regulation on edema is discussed in the section on the glymphatic system.

Aquaporin, a water channel highly polarized to paravascular endfeet, provides support for paravascular CSF–ISF exchange and drives the clearance of bulk interstitial solutes from brain parenchyma (Iliff et al., 2012). Some brain disorders, such as cerebral infarction, SAH, and traumatic brain injury, reduce polarized localization at the endfeet of reactive astrocytes (Wang et al., 2012; Ren et al., 2013; Fang et al., 2020). The abnormal distribution of AQP4 impairs the clearance of solute from cerebral parenchyma (Pu et al., 2019). AQP4 contributes to the spreading of edema after MCAO by facilitating the transport of CSF into the brain (Mestre et al., 2020). This pathophysiological process is in line with evidence demonstrating that AQP4-deficient mice and wild types treated with AQP4 inhibitors progress with less cerebral edema after ischemic stroke (Manley et al., 2000; Igarashi et al., 2011; Yao et al., 2015; Hirt et al., 2017; Pirici et al., 2018). Furthermore, the accelerated influx of water into the brain and elevated ICP in AQP4-overexpressing mice induced by intraperitoneal water injection confirm that the water channel protein promotes the occurrence of cerebral edema by increasing the permeability of the BBB (Yang et al., 2008). The function of AQP4 to control water uptake across BBB also reflects that up-regulated AQP4 can promote the formation of cerebral edema (Haj-Yasein et al., 2011). However, the function of astroglial AQP4 to drive the clearance of interstitial solutes from the brain parenchyma also illustrates that AQP4 may facilitate the absorption of excess fluid in brain edema (Iliff et al., 2012). Notably, much experimental evidence demonstrates the role of AQP4 in the resolution of brain edema (Papadopoulos et al., 2004; Finnie et al., 2008; Tourdias et al., 2009; Badaut et al., 2011a). In a model of vasogenic brain edema, AQP4-deficient mice have a significantly higher increase in ICP and brain water content compared with wild-type mice (Papadopoulos et al., 2004).

Interestingly, in contrast with animal brain models of ischemic stroke, we did not find similar results of cerebral edema in SAH (Luo et al., 2016; Liu et al., 2020). On the one hand, AQP4-deficient mice did not develop better neurological function and less neuroinflammation at day 7 after SAH (Luo et al., 2016). On the other hand, AQP4 deletion in mice significantly increased the water content in the whole brain and aggravated the neurological deficit following SAH; the possible mechanism involves AQP4 knockout impairing the glymphatic system function of facilitating ISF drainage to eliminate toxic factors in the brain (Tait et al., 2010; Liu et al., 2020). The increased vasogenic edema caused by AQP4 deletion is also found in other models of BBB disruption, including status epilepticus, brain tumor, and brain abscess (Bloch et al., 2005; Lee et al., 2012; Vella et al., 2015).

In conclusion, AQP4 plays a dual role in the process of cerebral edema after stroke with a harmful role in the early stages of edema formation and plays a beneficial role during edema subsidence (Badaut et al., 2011b; Vella et al., 2015; Clément et al., 2020). We believe that the role of AQP4 is closely related to its location in astrocytes and its supporting role in the glymphatic system. Therefore, controlling the function of AQP4 is a potential effective target for treating post-stroke cerebral edema, although there is still a lot of research work to be done.

## Meningeal Lymphatics

We have discovered that tracers injected into the brain parenchyma and ISF pass into the CSF and further into deep cervical lymph nodes (CLNs) (Koh et al., 2005; Iliff et al., 2012; Plog et al., 2015). However, it was not until 2015 that we found the basic structure of the metastatic pathway through the discovery of meningeal lymphatic vessels in mice (Aspelund et al., 2015). In the human brain, we also provided *in vivo* evidence of CSF tracer drainage to CLNs via meningeal vessels and that tracer enhancement within lymph nodes parallels glymphatic enhancement (Eide et al., 2018). Therefore, the discovery of the classical lymphatic drainage system in meninges also promotes us to think about its role in cerebral edema.

### Anatomical Considerations of the Meningeal Lymphatic System

Meningeal lymphatic vessels were discovered by chance after whole-mount mouse brain meninges stained by immunohistochemistry for different cells were used to determine the gateways responsible for T cells into and out of the meninges (Louveau et al., 2015). They found that high concentrations of T lymphocytes were abluminal and aligned linearly in CD31-expressing structures along sinuses (Louveau et al., 2015). These structures express markers of the lymphatic system, such as lymphatic vessel endothelial hyaluronan receptor-1, podoplanin, Prospero homeobox protein 1, VEGF receptor 3, CD31, and chemokine (C-C motif) ligand 21 (CCL21) (Aspelund et al., 2015; Louveau et al., 2015). Structurally, the meningeal lymphatic vessels are close to initial lymphatic capillaries but are devoid of SMCs, positive for the immune-cell chemoattractant protein CCL21, the punctate expression pattern of Claudin-5 and vascular endothelial cadherin, and the lack of integrin-a9 expression (Louveau et al., 2015). The lymphatic vessels accompany arteries and veins in the meninges, including the transverse sinus, sigmoid sinus, retroglenoid vein, superior sagittal sinus, rostral rhinal vein, and the major branches of the middle, anterior meningeal arteries (Aspelund et al., 2015), and meningeal septae penetrating the cerebral cortex (Lohrberg and Wilting, 2016). Lymphatic vessels can also be seen followed by the olfactory (CN I), optic (CN II), trigeminal (CN V), glossopharyngeal (CN IX), vagus (CN X), and accessory (CN XI) nerve sheaths (Aspelund et al., 2015; Absinta et al., 2017; Antila et al., 2017) and exit the skull along with CN IX, X, and XI (Aspelund et al., 2015). Except for CN IX, X, and XI, lymphatic vessels are also observed to exit the skull along the meningeal portions of the pterygopalatine artery, sigmoid sinus, and retroglenoid vein (Figure 3; Aspelund et al., 2015). The mechanism for the emergence of lymphatic vessels from the skull has yet to be discovered. The newly discovered meningeal lymphatic vessels constitute the second part of the current relatively complete CNS lymphatic drainage system. First, GSF flows into the brain interstitium along arterial perivascular spaces and is then cleared along paravenous drainage pathways to the CSF (Rasmussen et al., 2018). The last step is draining the CSF along the meningeal lymphatic vessels into the CLNs and communicating with the periphery. Therefore, the meningeal lymphatic system and the glymphatic system constitute a relatively complete cerebral lymphatic drainage system.

### Meningeal Lymphatic Involved in Edema Formation

More than two decades ago, some researchers have systematically studied the effects of cervical lymphatic blockade (CLB) in conditions such as ischemic stroke and SAH; the specific mechanisms by which CLB exerts an influence on stroke lesions remain unclear (Xing et al., 1994; Xia et al., 2003; Sun et al., 2000, 2006, 2009, 2011; Li et al., 2005, 2007; Si et al., 2006; Zheng et al., 2008). Before meningeal lymphatic vessels were discovered, cellular and soluble constituents of CSF were thought to enter the lymphatic vessels in brain mucosa through the cribriform lamina to elicit immune responses in CLNs (Weller et al., 2010; Laman and Weller, 2013; Mathieu et al., 2013). Now, studies have confirmed that meningeal lymphatic vessels, in addition to taking up and draining CSF, also directly communicate with CLNs to regulate intracranial inflammatory processes (Louveau et al., 2015; Dave et al., 2018; Ahn et al., 2019). Therefore, CLB not only directly leads to the disruption of meningeal lymphatic drainage, leading to intracranial hypertension and cerebral edema, but may also affect neuroinflammation by affecting the connection between the brain and the peripheral immune system (Sun et al., 2018). We can indirectly evaluate the role of meningeal lymphatic vessels in the entire process of edema occurrence and resolution by observing the effect of CLB on post-stroke edema.

In an ischemic stroke model, Sun et al. (2000) and Si et al. (2006) randomly assigned mice to the MCAO group and MCAO plus CLB group to determine the major effects of CLB (Sun et al., 2000; Si et al., 2006). In their experiments, they all observed that CLB aggravates brain edema caused by MCAO, which can be indicated by the content of water, sodium, calcium, and glutamate (Sun et al., 2000; Si et al., 2006). Compared with the MCAO only group, CLB + MCAO mice show decreased superoxide dismutase activity and a more markedly increased malondialdehyde content, which may indicate that CLB can deteriorate ischemic brain damage by promoting oxidative stress damage (Sun et al., 2000). Furthermore, the cerebral infarction volume and mRNA expression levels of N-methyl-D-aspartame receptor 1 in the ischemic hemisphere are markedly higher in rats with MCAO + CLB than in those with only MCAO at different time points (Si et al., 2006). CLB was also found to aggravate cerebral edema in a SAH model. After infusing arterial blood into the cisterna magna of mice to establish an experimental model of SAH with and without CLB, investigators found that regional CBF drops more obviously, and the increased ICP and brain water content were more serious in SAH plus CLB groups (Sun et al., 2006).

Many experiments have directly expounded the important role of the meningeal lymphatic system in the post-stroke activation of the processes of brain drainage and edema clearing (Chen et al., 2019; Semyachkina-Glushkovskaya et al., 2020; Yanev et al., 2020). The increase in the diameter of meningeal lymphatic vessels has been observed in a variety of experimental models, including cerebral hemorrhage, SAH, and the opening of the BBB (Semyachkina-Glushkovskaya et al., 2017, 2018, 2019, 2020). For example, only during the opening of the BBB can optical coherence tomography allow the observation of meningeal lymphatic vessels with increased diameter (Semyachkina-Glushkovskaya et al., 2017). Furthermore, the increased diameter of meningeal lymphatic vessels suggests the activation of meningeal lymphatic drainage function after stroke (Semyachkina-Glushkovskaya et al., 2020). Compared with the slow and non-remarkable accumulation of gold nanorods in the deep CLNs of normal mice, the extensive accumulation of gold nanorods in cavities of deep CLNs within three hours after SAH indicated the activation of lymphatic clearance as SAH progressed (Semyachkina-Glushkovskaya et al., 2019). Recent work also demonstrates that the increased outflow rate of meningeal lymphatics participates in the clearance of extravasated erythrocytes from CSF into CLNs after SAH (Chen et al., 2020). One week after SAH, long-term meningeal lymphatic clearance was proven to be dysfunctional (Pu et al., 2019).

In addition to cerebral hemorrhage and SAH, meningeal lymphatic drainage also plays a role in the pathophysiology of ischemic stroke (Chen et al., 2019; Yanev et al., 2020). Pavel Yanev and his colleagues found that meningeal lymphatic vessels sprouted from an adjacent sinus into the anatomical area corresponding to stroke in a mouse ischemic stroke model induced by photothrombosis; however, they detected no lymphangiogenesis in the tMCAO model (Yanev et al., 2020). Coincidentally, in a zebrafish ischemic stroke model induced by photochemical thrombosis, meningeal lymphatic cells rapidly grew into the injured brain parenchyma (Figure 3; Chen et al., 2019). These ingrown meningeal lymphatic vessels played a role in resolving cerebral edema and guiding and supporting the growth of nascent blood vessels (Chen et al., 2019). The role of lymphangiogenesis in promoting the regression of edema has also been confirmed in myocardial infarction (Vieira et al., 2018). Meningeal lymphatic hypoplasia was found to exacerbate stroke severity by increasing infarct size and causing sustained motor deficits in the tMCAo model (Yanev et al., 2020). Meningeal lymphatic dysfunction slows the efflux of macromolecules from the brain parenchyma (Da Mesquita et al., 2018). Furthermore, preexisting meningeal lymphatic dysfunction leads to aggravated neuroinflammation and cognitive outcomes following traumatic brain injury (Bolte et al., 2020).

In general, the meningeal lymphatic system plays a neuroprotective function after stroke and promotes the resolution of edema. Its function may mainly depend on two aspects. On the one hand, in the early stage of stroke, the activation of meningeal lymphatic drainage function can remove excess fluid in the skull; on the other hand, meningeal lymphatic vessels directly invade the injured brain parenchyma to resolve edema. The augmentation of lymphogenesis by treatment with VEGF-C improves heart function following myocardial infarct in mice (Klotz et al., 2015). VEGF-C also stimulates the drainage of meningeal lymphatic vessels in aged mice, resulting in improved cognitive function (Da Mesquita et al., 2018). Therefore, promoting the growth of meningeal lymphatic vessels seems to be beneficial for cerebral edema resolution and tissue repair.

### Meningeal Immunity Is Involved in Edema Formation

The discovery of meningeal lymphatic vessels has led to a collapse of dogma that the brain was an “immune-privileged” site, and its function to carry both fluid and immune cells from the CSF to the deep CLNs sets off an upsurge of how lymph nodes participate in the CNS immune response (Aspelund et al., 2015). Neuroinflammation following ischemic stroke plays a pivotal role in the breakdown of BBB, leading to vasogenic edema formation, hemorrhagic transformation, and aggravated patient prognosis (Rosenberg and Yang, 2007; Khatri et al., 2012). After focal cerebral ischemia in rats, the elevation of VEGF-C in CSF can increase pro-inflammatory macrophages by activating the lymphatic endothelium. By blocking VEGF-C/VEGFR3 signaling in lymphatic ECs, cytokine/chemokine expressions in superficial CLNs and pro-inflammatory macrophages in the ischemic area are significantly decreased, and the final effect is an obvious reduction in cerebral infarction volume (Esposito et al., 2019). This article focused on the aggregation and activation of macrophages during stroke, which is regulated by VEGF-C/VEGFR3 signaling in lymphatic ECs. However, the lymphocytes in the CLNs involved in the immune damage of stroke also include T and B cells, and many factors, such as neuropilin-2 (Xu et al., 2010), angiopoietins (Alitalo, 2006), BMP9-ALK1 (Yoshimatsu et al., 2013), DAMPs (Shichita et al., 2017), may participate in the activation of lymphatic endothelium after stroke. The function of meningeal lymphatic network in controlling immune responses in the CNS was also proved in a mouse model of glioblastoma (Song E. et al., 2020). The increased meningeal lymphatic drainage via VEGF-C can promote the priming of CD8 T cells in the draining CLNs and antitumor T cell responses (Song E. et al., 2020). Furthermore, the surgical and pharmacological blockade of meningeal lymphatic function diminishes the migration of activated encephalitogenic T cells into the CNS in an animal model of multiple sclerosis (Louveau et al., 2018). The presence of meningeal lymphatic vessels provides a link between the CNS and the peripheral immune system and provides a new therapeutic target for reducing neuroinflammation after stroke. However, the mechanism by which CLNs and meningeal lymphatic vessels participate in the immune response after stroke remains to be discovered.

## Conclusion

Traditionally, the occurrence of cerebral edema can be divided into three distinct phases: an early cytotoxic phase, a middle ionic phase, and a later vasogenic phase. Cytotoxic edema occurs within minutes after ischemic insult and ionic edema is the form of cerebral edema which forms immediately following cytotoxic edema and before barrier breakdown not occurring until 4–6 h after the onset of ischemia. Vasogenic edema, which is characterized by the breakdown of the BBB, manifests hours after the initial insult. However, the discovery of the glymphatic system and meningeal lymphatic vessels adds new content to each phase. Ion transporter dysfunction at the NVU lays the foundation for cytotoxic edema and ionic edema, and GSF, which flows rapidly into brain parenchyma through the glymphatic system within minutes after insult, also acts as the source of edema fluid. As the primary initial event driving tissue swelling, the glymphatic inflow of CSF may provide a basis for the treatment of cerebral edema after stroke. The breakdown of the BBB, which results in vasogenic edema formation, involves many joint effects, including the destruction of TJs, imbalance of the NVU, damage of inflammatory response, and activation of ion channels. Inflammation is a key element among many factors that lead to the progression of BBB damage in stroke. The glymphatic system plays a dual role in the process of cerebral edema after stroke with a harmful role in the early stage of edema formation and plays a beneficial role during edema subsidence. The function of glymphatic system is supported by astrocytic AQP4. AQP4 can not only control water flux across the BBB, but also facilitate the circulation of CSF, and drive the clearance of bulk interstitial solutes from the brain parenchyma. Therefore, in the earlier phases of cerebral edema (cytotoxic and ionic edema), the up-regulation of AQP4 can aggravate brain edema formation, while in the vasogenic edema phases, AQP4 may play a key role in the elimination of water of vasogenic origin. The activation of meningeal lymphatic drainage function and the ingrown of meningeal lymphatic vessels into the injured brain parenchyma promote the resolution of edema after stroke. Consequently, the rigorous dissection of the pathophysiology of the cerebral lymphatic system may eventually lead us to novel mechanisms and targets for cerebral edema after stroke.

## Author Contributions

SC and LM collected information and drafted and revised the manuscript. LS contributed to collecting information and editing the manuscript. LM directed the work and finalized the manuscript. All authors agreed to be accountable for the content of the work.

## Conflict of Interest

The authors declare that the research was conducted in the absence of any commercial or financial relationships that could be construed as a potential conflict of interest.

## Publisher’s Note

All claims expressed in this article are solely those of the authors and do not necessarily represent those of their affiliated organizations, or those of the publisher, the editors and the reviewers. Any product that may be evaluated in this article, or claim that may be made by its manufacturer, is not guaranteed or endorsed by the publisher.
